# Crystal structure and ligand-induced folding of the SAM/SAH riboswitch

**DOI:** 10.1093/nar/gkaa493

**Published:** 2020-06-10

**Authors:** Lin Huang, Ting-Wei Liao, Jia Wang, Taekjip Ha, David M J Lilley

**Affiliations:** Guangdong Provincial Key Laboratory of Malignant Tumor Epigenetics and Gene Regulation, Medical Research Center, Sun Yat-Sen Memorial Hospital, Sun Yat-Sen University, Guangzhou 510120, P. R. China; RNA Biomedical Institute, Sun Yat-Sen Memorial Hospital, Sun Yat-Sen University, Guangzhou 510120, P. R. China; Cancer Research UK Nucleic Acid Structure Research Group, MSI/WTB Complex, The University of Dundee, Dow Street, Dundee DD1 5EH, UK; Department of Biophysics; Cancer Research UK Nucleic Acid Structure Research Group, MSI/WTB Complex, The University of Dundee, Dow Street, Dundee DD1 5EH, UK; Department of Biophysics; Department of Biophysics and Biophysical Chemistry; Department of Biomedical Engineering, Johns Hopkins University, Baltimore, MD 21205, USA; Howard Hughes Medical Institute, Baltimore, MD, USA; Cancer Research UK Nucleic Acid Structure Research Group, MSI/WTB Complex, The University of Dundee, Dow Street, Dundee DD1 5EH, UK

## Abstract

While most SAM riboswitches strongly discriminate between SAM and SAH, the SAM/SAH riboswitch responds to both ligands with similar apparent affinities. We have determined crystal structures of the SAM/SAH riboswitch bound to SAH, SAM and other variant ligands at high resolution. The riboswitch forms an H-type pseudoknot structure with coaxial alignment of the stem–loop helix (P1) and the pseudoknot helix (PK). An additional three base pairs form at the non-open end of P1, and the ligand is bound at the interface between the P1 extension and the PK helix. The adenine nucleobase is stacked into the helix and forms a *trans* Hoogsteen–Watson–Crick base pair with a uridine, thus becoming an integral part of the helical structure. The majority of the specific interactions are formed with the adenosine. The methionine or homocysteine chain lies in the groove making a single hydrogen bond, and there is no discrimination between the sulfonium of SAM or the thioether of SAH. Single-molecule FRET analysis reveals that the riboswitch exists in two distinct conformations, and that addition of SAM or SAH shifts the population into a stable state that likely corresponds to the form observed in the crystal. A model for translational regulation is presented whereby in the absence of ligand the riboswitch is largely unfolded, lacking the PK helix so that translation can be initiated at the ribosome binding site. But the presence of ligand stabilizes the folded conformation that includes the PK helix, so occluding the ribosome binding site and thus preventing the initiation of translation.

## INTRODUCTION

Control of gene expression by binding of small-molecule metabolites directly to an upstream untranslated region of mRNA is widespread in bacteria (1–3). In these riboswitches, the local RNA structure creates a specific binding site for the small-molecule ligand that stabilizes an alternative structure in the RNA that alters the rate of either transcription or translation of the adjacent gene. Riboswitches can function as either OFF or ON switches, i.e. gene expression is reduced or increased on binding the ligand. In general, the effector ligand is a metabolite such as the substrate or product of the protein expressed from the gene that is controlled, or of another enzyme in the same metabolic pathway. Approximately 40 classes of riboswitches have been identified to date, a significant fraction of which bind coenzymes.

**Figure 1.**
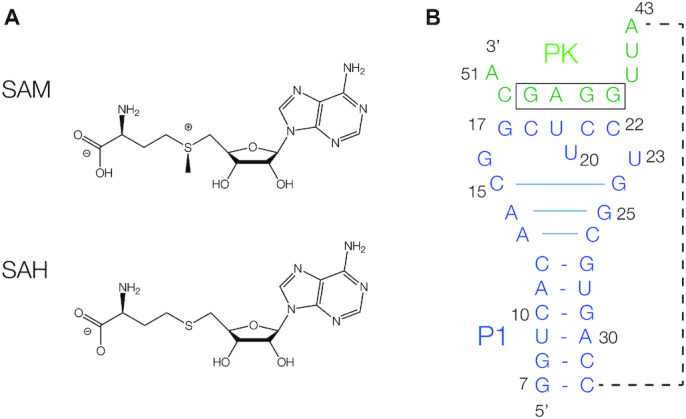
The ligands and secondary structure of the SAM/SAH riboswitch. (**A**) The chemical structures of SAM and SAH. (**B**) The secondary structure of the SK209-52 SAM/SAH riboswitch in its pseudoknot conformation. The 5′ end of the molecule (blue) can adopt a stem-loop structure (P1). The 3′ end (green) can base pair with the center of the loop to generate five Watson–Crick base pairs (PK), thus forming an H-type pseudoknot. Three additional base pairs form at the top of the P1 helix, indicated by the longer horizontal lines. The Shine–Dalgarno ribosome binding site (boxed) is an integral part of the PK helix. We have followed the numbering scheme used by Weinberg *et al.* (16), so that the 5′ nucleotide is position 7 in the sequence. The oligonucleotide chain connecting the 3′-end of the P1 helix to the 5′ end of the PK helix (shown by the broken line) is not present in our crystallographic construct.

Together with the thiamine pyrophosphate-binding riboswitches, one of the largest group of riboswitches are those that bind the coenzyme *S*-adenosylmethionine (SAM; Figure 1A) (4). SAM is used by methyltransferase enzymes as a donor of a methyl group to many and diverse substrates (5–7). The methyl group is directly bonded to the sulfur atom, which is therefore a sulphonium cation. On donation of the methyl SAM is converted to S-adenosylhomocysteine (SAH; Figure 1A), an electrically-neutral thioether that is toxic in the cell (8,9).

A number of distinct classes of riboswitch bind SAM (10,11), which can be classified into three families, and structures are available for each. The first to be discovered was the SAM-I riboswitch (12–14), and subsequently the SAM-IV (15), SAM-I/IV (16) and SAM-I (12,13) were identified as members of the same family. The SAM-II riboswitch (17) is the basis of the second family, which also includes the SAM-V riboswitch (18). SAM-II and SAM-V riboswitches are based on a pseudoknot architecture (19,20). Lastly, the SAM-III riboswitch (21) uses a three-way helical junction to create the SAM binding site; (22). The SAM-VI riboswitch has been classed within the SAM-III family (11), although a recent structural study suggests that this riboswitch should be classified on its own (23). In addition to these riboswitches, a SAH-binding riboswitch has also been identified (24).

RNA is a very adaptable ligand for small molecules, exemplified both by the riboswitches and *in vitro*-selected aptamer species (25,26). In general, riboswitches exhibit great discrimination in the binding of their target molecule, and the exclusion of chemically similar species. For example, the guanidine riboswitches bind guanidine but exclude urea, that differs only by the exchange of one amine for a carbonyl group (27–29). They do this in part by having a binding site that lacks hydrogen bond donors, as well as employing a cation-π interaction that discriminates the charged guanidinium cation from the electrically-neutral urea (30–33). The SAM riboswitches bind their ligand in a variety of ways and in different conformations that will be discussed below, yet in general they discriminate strongly against the binding of SAH. The principal difference between the two species is the charge on the sulfur atom. This can be tested by juxtaposing one or more uracil carbonyl oxygen atoms that carry a significant partial negative charge. This is seen particular clearly in the case of the SAM II (19) and SAM-V (20) riboswitches. In both riboswitches two uracil nucleobases are oriented so that the electrical dipoles of the exocyclic carbonyl groups at the 4 position are directly oriented toward the sulfonium, with the oxygen atoms located just 3.2 Å away. By contrast to the SAM riboswitches, the SAH riboswitch has the inverse specificity with a 1000-fold discrimination of SAH over SAM (34). The structure of this riboswitch suggests that the selectivity for SAH is mediated primarily by a steric clash when a methyl group is bonded to the sulfur (35).

The SAM/SAH riboswitch was identified in the order Rhodobacterales of α-proteobacteria by the Breaker lab (16). Bioinformatic analysis of 552 novel SAM/SAH riboswitches obtained from Rfam found that almost all were associated with the SAM synthetase gene *met*K (36). In contrast to the SAM and SAH riboswitches, the SAM/SAH riboswitch has a relaxed specificity and binds both SAM and SAH with similar affinity in the low micromolar region. This then provides an interesting problem in molecular recognition. An earlier NMR study by Wöhnert and colleagues (36) provided a solution structure of the SAM/SAH riboswitch bound to SAH, showing that it formed the expected pseudoknot structure. In this study we have solved the crystal structure of the riboswitch bound to SAM, SAH and four other ligands. We have also performed single-molecule FRET studies to examine structural changes on binding ligand, leading to a proposed mechanism of action of the riboswitch.

## MATERIALS AND METHODS

### Riboswitch ligands

SAM (A7007), SAH (A9384), AMP (A1752), adenosine (A9251), 5′-deoxy-5′-(methylthio)adenosine (D5011) and decarboxy-SAH (43713) were all obtained from Sigma.

### Sequence alignment and analysis

The SAM/SAH riboswitch sequences were taken from Rfam under accession RF01727. The sequences were visualized and analysed by Jalview (37), and several RNA sequences were selected for crystallization trials based on length and conservation. All sequence analysis was performed in Jalview, using the published alignment.

### RNA synthesis

The SAM/SAH riboswitch sequence was derived from *Roseobacter* sp. SK209-2-6. All sequences are written 5′ to 3′.

SK209-50 RNA used for calorimetry: UACCUGUCACAACGGCUUCCUGGCGUGACGAGGUGACCUCAGUGGAGCAA

The wild type sequence, the U23C mutant and atomic mutants were derived from this sequence.

Crystallization:

The crystallized construct was generated by the hybridization of two oligonucleotides (^Br^C = 5-bromocytidine):

RNA1-26: GGU ^Br^CACAACGGCUUCCUGGCGUGACC

RNA2-9: AUUGGAGCA

Singe-molecule FRET analysis:

The SAM/SAH riboswitch for single molecule measurements contains a Cy3 fluorophore attached to the O2′ of U20 generated by Cu^2+^-catalyzed reaction of alkyne-modified RNA with an azide-attached fluorophore (Lumiprobe Corp). The RNA had an 18 nt 3′ DNA extension for base-pairing to the anchor DNA, and the complete sequence was (DNA underscored):

GAUACCUGUCACAACGGCU(U-Cy3)CCUGGCGUGACGAGGUGACCUCAGUGGAGCAA ACCGCTGCCGTCGCTCCG

The anchor DNA had a 5′-biotin and 3′ Cy5 fluorophore, and was complementary to the 18 nt extension of the SAM-SAH riboswitch strand. Its sequence was:

biotin-CGGAGCGACGGCAGCGGT-Cy5

RNA oligonucleotides were synthesized using ***t***-BDMS phosphoramidite chemistry (38) as described in Wilson *et al.* (39), implemented on an Applied Biosystems 394DNA/RNA synthesizer. RNA was synthesized using ribonucleotide phosphoramidites with 2′-*O*-*tert*-butyldimethyl-silyl (*t*-BDMS) protection (40,41) (Link Technologies). Oligonucleotides containing 5-bromocytidine (ChemGenes) were deprotected in a 25% ethanol/ammonia solution for 36 h at 20°C. All oligoribonucleotides were redissolved in 100 μl of anhydrous DMSO and 125 μl triethylamine trihydrofluoride (Sigma-Aldrich) to remove *t*-BDMS groups, and agitated at 65°C in the dark for 2.5 h. After cooling on ice for 10 min, the RNA was precipitated with 1 ml of butanol, washed once with 70% ethanol and suspended in double-distilled water.

RNA was further purified by gel electrophoresis in polyacrylamide under denaturing conditions in the presence of 7 M urea. The full-length RNA product was visualized by UV shadowing. The band was excised and electroeluted using an Elutrap Electroelution System (GE Healthcare) into 45 mM Tris-borate (pH 8.5), 5 mM EDTA buffer for 12 h. at 150 V at 4°C. The RNA was precipitated with isopropanol, washed once with 70% ethanol and suspended in water or ITC buffer (40 mM HEPES-K (pH 7.0), 100 mM KCl, 10 mM MgCl_2_).

### Crystallization, structure determination and refinement

A solution of 1 mM RNA1-26 was mixed with 1.1 mM RNA2-9 in 5 mM HEPES (pH 7.6), 100 mM KCl and the mixture heated to 95°C for 1 min. The solution was slowly cooled to 20°C and MgCl_2_ added to a final concentration of 5 mM. Ligands (Sigma-Aldrich) were added to a final concentration of 5 mM. Crystals were grown by sitting drop vapor diffusion at 20°C using drops prepared by mixing 1 μl of the RNA–ligand complex with 1 μl of a reservoir solution comprising 0.01 M magnesium sulfate, 0.05 M sodium cacodylate (pH 6.0–6.6) and 1.8 M lithium sulfate monohydrate. All structures were obtained by co-crystallization with the six different ligands. Crystals appeared after 3 weeks. The crystals were transferred into mother liquid with 30% extra glycerol, then flash frozen by mounting in nylon loops and plunging into liquid nitrogen.

Diffraction data were collected on beamline I04, I04-1 or I24 at Diamond Light Source (Harwell, UK). Data were processed by XIA2 (42). The resolution cutoff for the data was determined by examining by CC1/2 and the density map (43). The structure was determined by Br-SAD by AutoSol in PHENIX suite, or molecular replacement using PHASER (44) with the search model 6YMM. Crystals grew in space group P312 with unit cell dimensions *a* = 88 Å, *b* = 88 Å and *c* = 76 Å or C2 with unit cell dimensions *a* = 87 Å, *b* = 148 Å and *c* = 75 Å (Supplementary Table S1). Models were adjusted manually using Coot (45) and subjected to several rounds of adjustment and optimization using Coot, phenix.refine and PDB_REDO (46). Composite omit maps were calculated using PHENIX. Model geometry and the fit to electron-density maps were monitored with MOLPROBITY (47) and the validation tools in Coot. Atomic coordinates and structure factor amplitudes have been deposited with the PDB with accession code as listed in Supplementary Table S1. Crystallographic statistics are presented in Supplementary Table S1.

### Isothermal titration calorimetry

Titrations were performed at 298 K using an ITC-200 microcalorimeter (GE). RNA solutions at 40 μM were prepared by diluting concentrated stocks into binding buffer containing 40 mM HEPES-K (pH 7.0), 100 mM KCl, 10 mM MgCl_2_ (ITC buffer). Ligands were prepared in the same binding buffer at a concentration of 500 μM. The sample cell was filled with 200 μl of RNA. Ligands were injected in a volume of 0.4 μl for the first injection and 2 μl for the next 19 injections using a computer-controlled 40 μl microsyringe with an injection interval of 120 s. Titration of ligands into the binding buffer or titration of the binding buffer into the RNA solution resulted in negligible evolution of heat. Integrated heat data were analyzed using a one-set-of-sites model in MicroCal Origin following the manufacturer's instructions. The first data point was excluded in analysis. The binding parameters enthalpy ΔH (cal mol^−1^), association constant *K*_a_ (M^−1^) and *n* (bound ligands per RNA) were variables in the fit. The binding free energy Δ*G* and reaction entropy ΔS were calculated using the relationships Δ*G*°  =  −*RT* ln *K*, where *R* = 1.987 cal mol^−1^ K^−1^, *T* = 298 K and Δ*G*° = Δ*H* − *T*Δ*S*. The dissociation constant *K*_d_ was calculated as 1/*K*_a_.

### Single-molecule FRET analysis

For annealing the sample for smFRET measurements, the SAM/SAH riboswitch molecules and the anchor DNA were annealed with molar ratio 1.5:1 under T50 (10 mM Tris (pH 8.0), 50 mM NaCl) buffer followed by slow cooling from 95°C to room temperature. 40 pM of the pre-annealed SAM/SAH riboswitch molecules were immobilized on a neutravidin-functionalized, polymer-passivated surface and free molecules were washed out with T50 buffer. Image buffer was freshly mixed before measurements, comprising 2.5 mM protocatechuic acid (PCA), 2.5 mM TSY (a triplet state quencher), and 50 nM protocatechuate-3,4-dioxygenase (PCD) enzyme in buffer containing 40 mM HEPES (pH 7.5), 100 mM KCl, 2 mM MgCl_2_. All the ligands were diluted with image buffer immediately prior to measurements. PCA, TSY, and PCD enzyme were purchased from Pacific Biosciences. The ligands were incubated for 5 min before imaging.

Single-molecule FRET data were obtained using a prism-based total internal reflection fluorescence (TIRF) microscope. The Cy3 and Cy5 fluorophores were excited by a 532-nm laser (Coherent Compass 315M) and a 638-nm laser (Cobolt 06-MLD) respectively. The fluorescence emission was collected by a water immersion objective (Olympus NA 1.2, 60×) and recorded by a back-illuminated electron-multiplying charge-coupled device camera (iXON, Andor Technology) with a dual-view setup. The dual-view setup used a long-pass emission filter (Semrock BLP02-561R-25) for eliminating the 532-nm laser, and a notch filter (Chroma ZET633TopNotch) for eliminating the 638-nm laser. The fluorescence emission was separated into donor and acceptor emission by a long-pass dichroic mirror (Semrock FF640-FDi01-25 × 36). The passivated PEG quartz slides and coverslips were purchased from Johns Hopkins Slides Core and were assembled into a reaction chamber (48). Spots detection, background subtraction, donor leakage and acceptor direct-excitation correction followed our previous protocol (48). All custom codes are available upon request.

For the three populations in the presence of no ligand, 1 mM SAM or 1 mM SAH, traces in excess of 50 s were collected and were categorized into constant high FRET, constant low FRET, or dynamic. For the dynamic population, the regions of dynamics were collected and analyzed by ebFRET (49) and the two-state dwell time was fitted to a single exponential function.

## RESULTS

### Crystallization of the SAM/SAH riboswitch

The RNA used in our crystallographic study was adapted from the sequence of the *Roseobacter* sp. SK207-2-6 studied by Weinberg *et al.* (16). The deduced secondary structure is that of a pseudoknot comprising P1 and PK helices (Figure 1). Our RNA comprised two segments, corresponding to the P1 stem-loop and the other strand of the PK helix; the section linking the 3′ end of P1 to the PK strand was therefore omitted from this construct. 5-bromocytidine was included at nucleotide 10 within P1 to provide phasing information. The RNA was co-crystallized with six different ligands and crystals were obtained diffracting to a resolution of 1.70–2.50 Å (Supplementary Table S1). The structure of the riboswitch bound to SAM was solved by SAD using the anomalous scatter of the bromine, and the other complexes were solved by molecular replacement using that structure.

### Crystal structure of the SAM/SAH riboswitch

**Figure 2.**
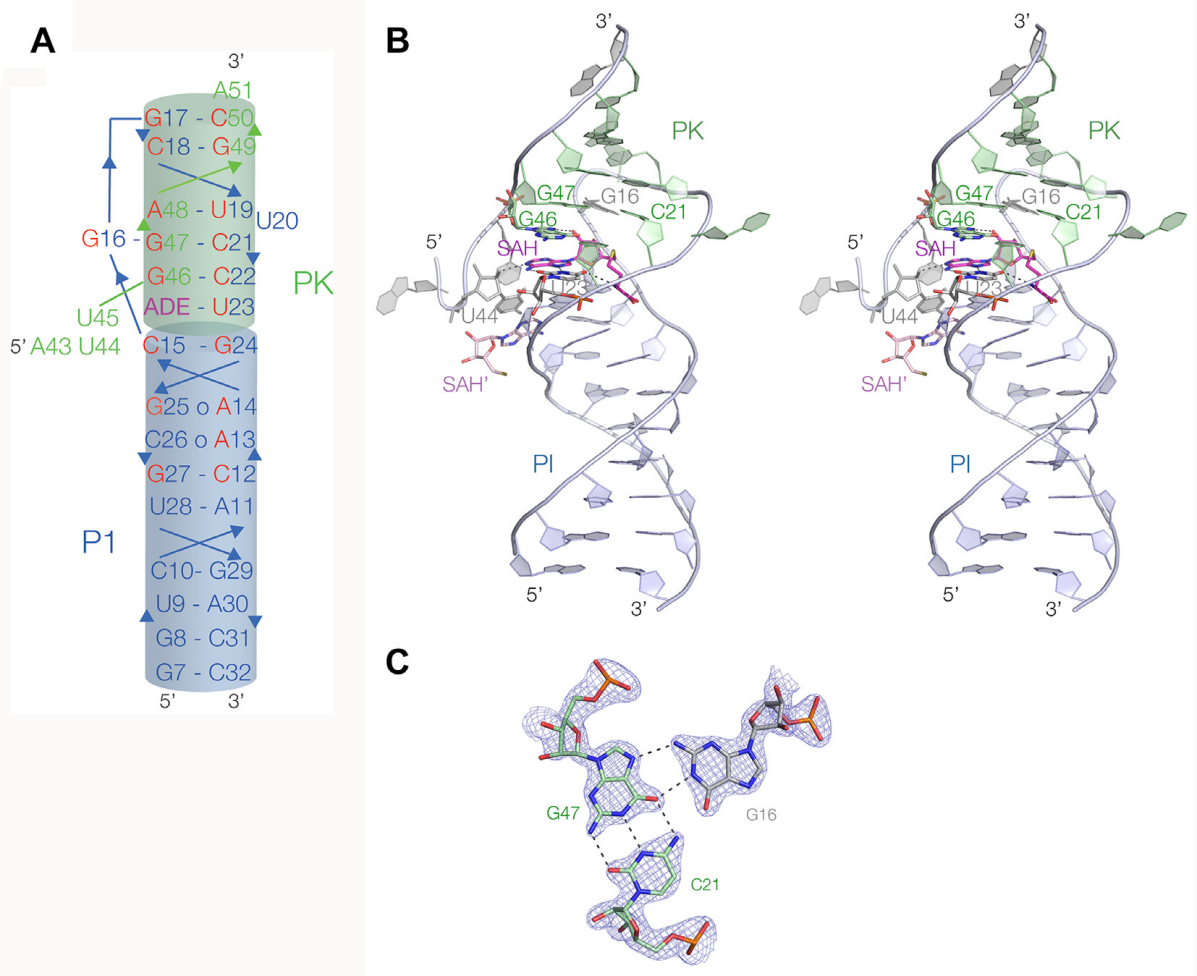
The structure of the SAM/SAH riboswitch in the crystal. This shows the overall structure of the riboswitch bound to SAH. (**A**) A schematic depicting the structure of the ligand-bound form of the riboswitch, illustrating the coaxial nature of the P1 and PK helices and showing the strand connectivity. Highly conserved nucleotides are colored red. (**B**) Parallel-eye stereoscopic view of the overall structure of the riboswitch bound to SAH in the crystal, at a resolution of 1.7 Å. There is coaxial alignment of the P1 helix (blue) and the PK helix (green). The main ligand binding site lies at the interface between the P1 and PK helices; the SAH is colored magenta. A second bound SAH molecule (SAH’ colored pale magenta) lies at the interface between two riboswitches, see Supplementary Figure S1. (**C**) The structure of the G16:G47–C21 base triple interaction, with the electron density from the omit map shown contoured at 2.5σ.

The SAM/SAH riboswitch adopts a standard H-type pseudoknot fold, with coaxial stacking between the P1 and PK helices (Figure 2A, B). In the C2 crystal lattice, six rod-like riboswitch molecules associate in the form of a triangle in the crystal lattice (Supplementary Figure S1A). The edges of the triangle are formed by coaxial stacking of the P1 helices of two RNA molecules, and the vertices of the triangle are formed by a crossing of the PK helices at 60°. The junction is not 2-fold symmetric, and A43 from one riboswitch molecule locates in the minor groove of the PK helix of the other.

At the base of the loop the P1 helix is extended by formation of three additional base pairs, i.e. A13: C26 (*cis* sugar-Hoogsteen), A14: G25 (*trans* Hoogsteen-sugar) and C15:G24 (standard *cis*-Watson–Crick). With the formation of these three loop-proximal base pairs of P1, the remaining loop then comprises G16 through to U23. Nucleotides G17 through to C22 form five *cis*-Watson–Crick base pairs with G46 through to C50 that define the PK helix, with the exception of U20 that is extruded out of the helix. At the 5′ end of the loop that forms the PK helix there is a sharp turn at G16; this forms a base triple with the C21:G47 base pair of PK, forming a *cis*-Watson-Crick-Hoogsteen base pair with G47 (Figure 2C). At the interface between P1 and PK, U23 pairs with the adenine of the ligand to form a base pair that is continuous with P1 and PK, discussed in more detail below. The sequence of this entire region is >97% conserved (16) (Supplementary Figure S2), and forms the ligand binding site. Nucleotides A43, U44 and U45 define the 3′ end of the linker between P1 and PK, the remaining part being absent in our structure. U44 and U45 are stacked on each other with their Watson-Crick edges directed into the minor groove of the helix at the P1–PK interface. U44 O2′ is hydrogen bonded to the ligand (see below) and U44 O2 accepts a hydrogen bond from G24 N2 of the P1 helix.

### SAH binding in the SAM/SAH riboswitch

**Figure 3.**
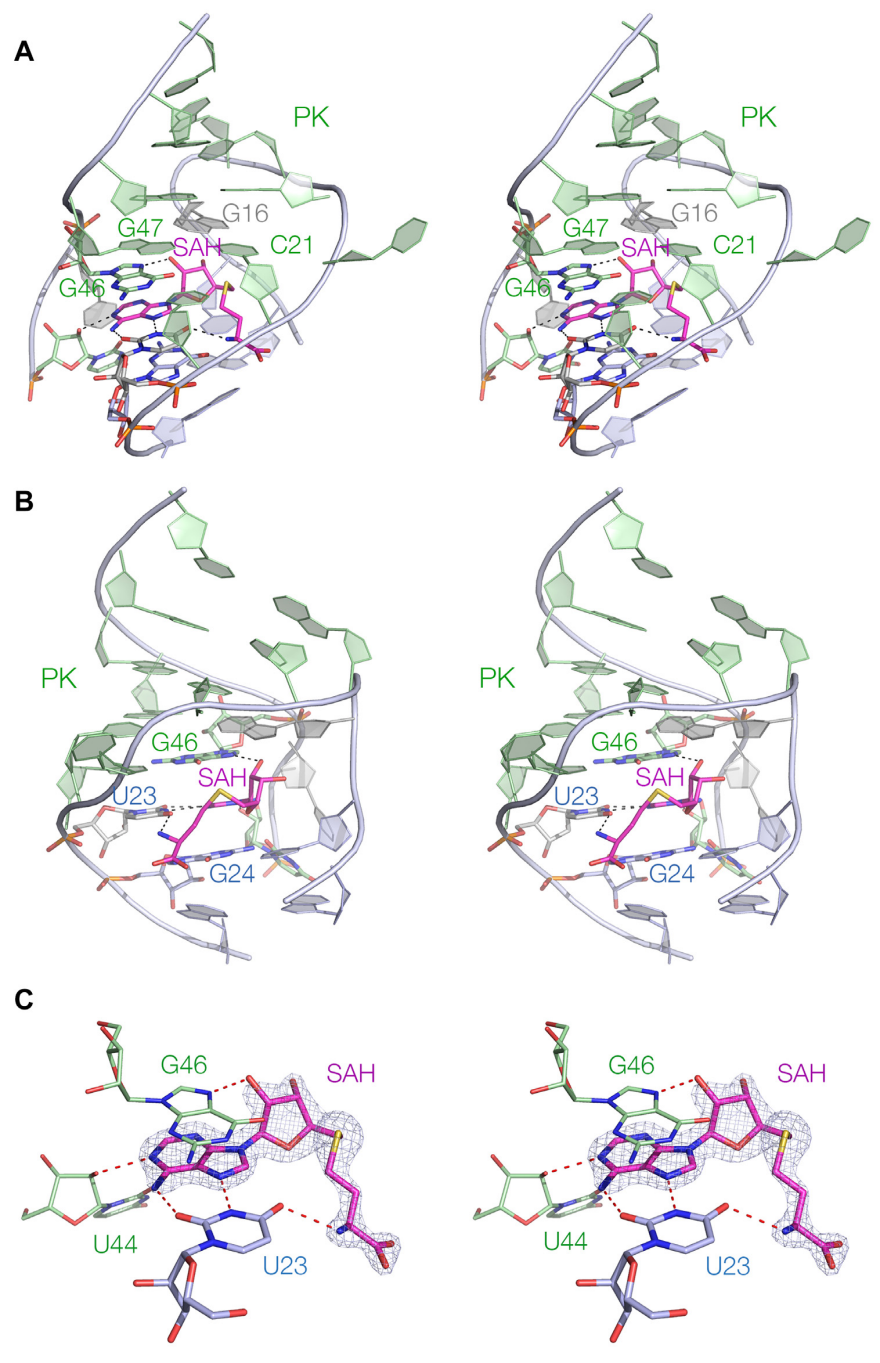
The ligand binding site of the SAM/SAH riboswitch. Parallel-eye stereoscopic views are shown of SAH bound to the riboswitch. (A, B) View of the bound SAH observed viewing into the minor groove of the PK helix (**A**) and viewed into the major groove side of the P1 loop (**B**). The SAH adenine nucleobase is base paired with U23, and stacked between G24 at the top of the extended P1 helix, and G46 in the PK helix. The hydrogen bonds connecting the ligand and the RNA are shown in red. The homocysteine chain of the ligand lies in the center of the loop and makes few interactions with the RNA. (**C**) Close-up view of the bound SAH showing the interactions with the riboswitch binding site. The omit electron density map of the SAH is shown contoured at 2.5 σ.

The SAH ligand is located at the interface between the P1 and PK helices. The adenine moiety adopts an *anti* conformation, and the nucleobase is stacked between G46 and G24 (Figure 3). It forms a *trans* Hoogsteen-Watson Crick base pair with U23 thus becoming an integral part of the coaxial P1-PK helices. The homocysteine chain is directed into the major groove of the P1 helix. The SAH adenine:U23 base pair is connected by two hydrogen bonds, i.e. adenine N6 to U23 O2 (2.9 Å) and U23 N3 to adenine N7 (2.7 Å). In addition, SAH adenine N1 accepts a hydrogen bond from U44 O2′ (2.7 Å), and the SAH O2′ donates one to G46 N7 (2.7 Å). Lastly the SAH methionyl amide N donates a hydrogen bond to U23 O4 (2.8 Å). Thus, altogether the SAH ligand forms five hydrogen bonds to the RNA that should substantially stabilize the structure of the PK helix.

**Figure 4.**
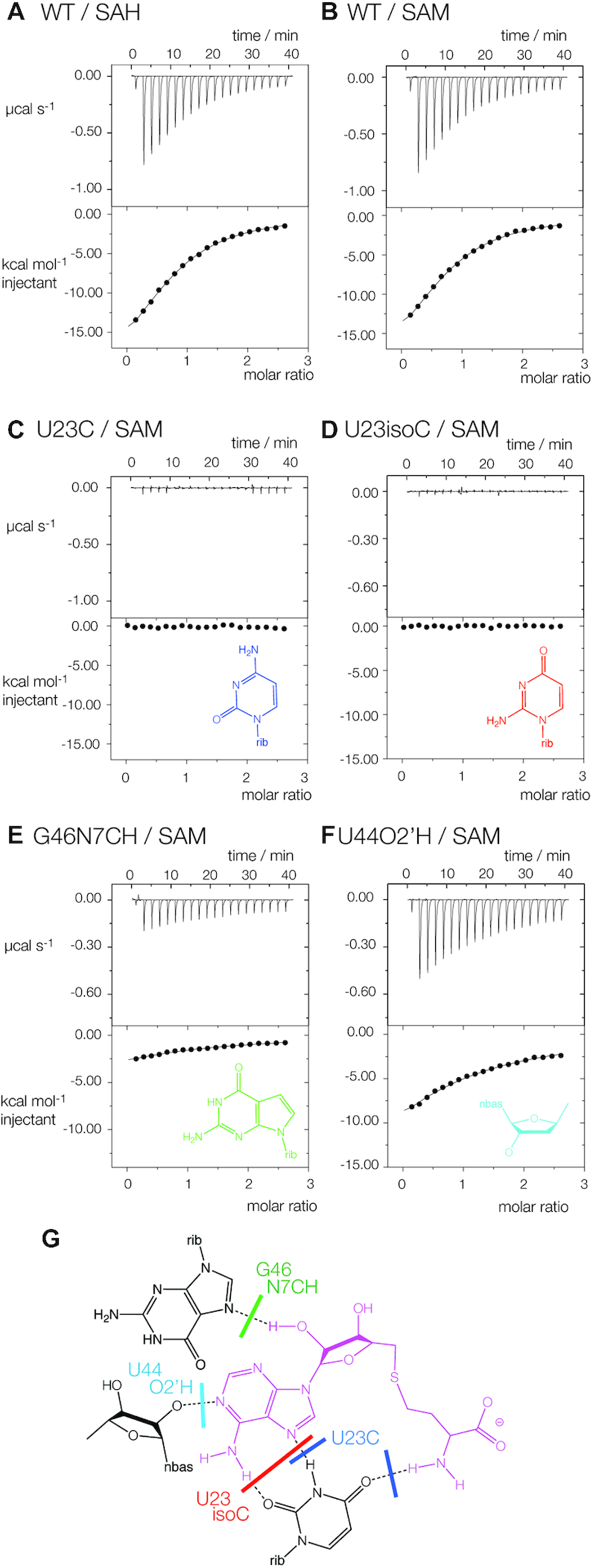
Analysis of ligand–RNA interactions in the SAM/SAH riboswitch using isothermal titration calorimetry and atomic mutagenesis. A solution of SAM or SAH was was titrated into a wild-type or variant SAM/SAH riboswitch solution, and the heat evolved was measured as the power required to maintain zero temperature difference with a reference cell. Integration over time gives the heat required to maintain thermal equilibrium between cells. In each case, the upper panel shows the raw data for sequential injections of 2 μl volumes (following an initial injection of 0.4 μl) of a 1 mM solution of ligands into 200 μl of a 40 μM RNA solution in 40 mM HEPES (pH 7.0), 100 mM KCl, 10 mM MgCl_2_. This represents the differential of the total heat (i.e. enthalpy Δ*H*° under conditions of constant pressure) for each ligand concentration. Integrated heat data were analyzed using a one-set-of-sites model in MicroCal Origin following the manufacturer's instructions. The first data point was excluded in the analysis. All ITC experiments were repeated a total of three times. The thermodynamic parameters calculated from these data are presented in Supplementary Table S3. The chemical structures of modified nucleotides are shown inset where appropriate. (**A**, **B**) Titration of (**A**) SAH and (**B**) SAM into unmodified SAM/SAH riboswitch RNA. (C–F) Titration of SAM into (**C**) U23C-modified, (**D**) U23isoC-modified, (**E**) G46N7CH-modified and (**F**) G44O2′H-modified SAM/SAH riboswitch RNA. (**G**) The chemical structure of the active site, showing the interactions that should be disrupted by the various atomic substitutions.

The importance of these contacts for ligand binding have been explored using atomic mutagenesis and isothermal titration calorimetry (ITC) (Figure 4). Both SAH (Figure 4A) and SAM (Figure 4B) resulted in exothermic binding of ligand with closely similar calculated affinities of *K*_d_ = 20 and 19 μM respectively. In contrast, all the atomic mutants studied led to impaired SAM binding. In view of the mode of binding we would expect that mutation of U23 would be especially critical. Substitution by either cytosine (Figure 4C, preventing hydrogen bond formation to SAM adenine N7 and the amide) or isocytosine (Figure 4D, preventing hydrogen bond formation to SAM adenine N6 and N7) resulted in no evolution of heat on titration of SAM, i.e. no detectable ligand binding. G46 N7CH led to virtually no detectable binding (Figure 4E), demonstrating the importance of the hydrogen bond donated by the O2′ of SAM. Lastly, removal of the O2′ from the nucleotide at position 44 greatly impaired SAM binding (Figure 4F), indicating the importance of the hydrogen bond donated to the SAM adenine N1.

We observed a second molecule of SAH bound within the crystal lattice (Supplementary Figure S1B). This is located at the apex of the triangular arrangement of riboswitch molecules, and is hydrogen bonded to both molecules. The SAH adenine makes three hydrogen bonds to one riboswitch, N6 to A14 N1, N1 to A14 N6 and N3 to G24 O2′. SAH O2′ is hydrogen bonded to the *pro*R non-bridging O of U44. The same SAH molecule is bound to the other riboswitch molecule at the lattice interface, its O3′ donating a hydrogen bond to the *pro*S non-bridging O of U23′. Thus the binding site is created by the close association of the two riboswitch molecules in the crystal lattice and is unlikely to exist in free solution. This is also consistent with the stoichiometry of binding measured by calorimetry (see below). We conclude that this mode of binding is not relevant to the cellular function of the riboswitch.

### Binding of SAM and variant ligands to the SAM/SAH riboswitch

**Figure 5.**
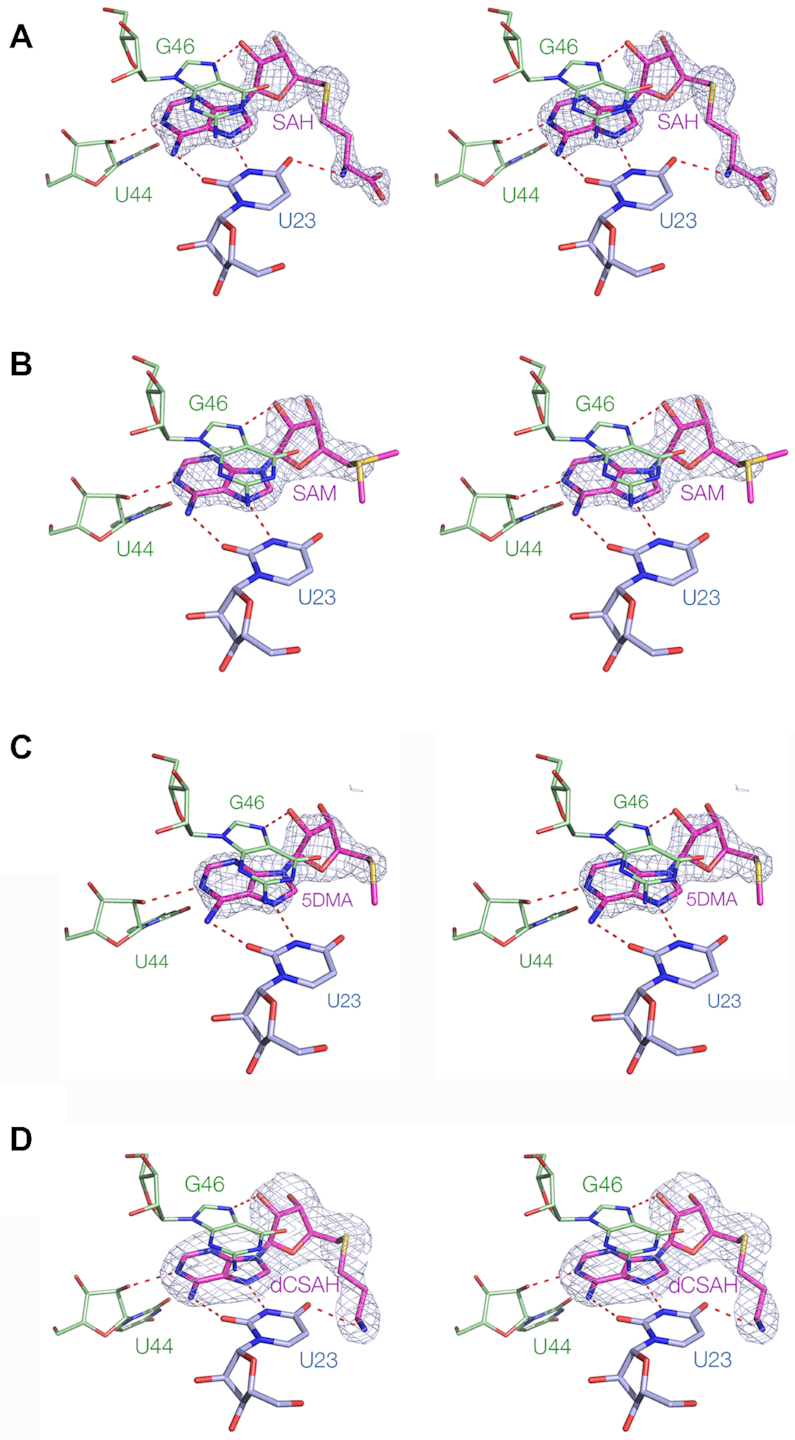
Crystal structures of the SAM/SAH riboswitch RNA bound to six different ligands. In each case a parallel-eye stereoscopic view of the binding site is shown, with the omit electron density map of the ligand contoured at 2.5σ. The ligands are (**A**) SAH, (**B**) SAM, (**C**) 5DMA and (**D**) dCSAH. Electron density for the methionine chain of SAM is not visible beyond the sulfur atom. The chemical structures of the ligands are shown in Supplementary Figure S3, and crystal structures for two further ligands are shown in Supplementary Figure S4.

We have also determined the structure of the SAM/SAH riboswitch bound by six different ligands, whose structures are shown in Supplementary Figure S3. All are based on adenosine, with variable chains attached to the 5′ position based on methionine [SAH and SAM, decarboxy-SAH (dCSAH), 5′-deoxy-5′-(methylthio)adenosine (5DMA)], phosphate (AMP) or hydroxyl (adenosine). The overall structures of the RNA is the same for each, and the structures of the ligand binding sites are shown in Figure 5 (SAH, SAM, dCSAH and 5DMA) and Supplementary Figure S4 (adenosine and AMP). The adenosine sections are bound identically in all six complexes, with the same four hydrogen bonds between the RNA and ligand in each case. The differences lie in the methionine section of the molecules. Where present the sulfur atoms are located in the same position, irrespective of whether they are positively charged (SAM) or neutral (SAH, dCSAH, 5DMA). Electron density for the remaining part of the methionine is not visible for the bound SAM ligand, but is observable for the bound dCSAH showing that the primary amine is hydrogen bonded to U23 O4 just as it is for SAH.

### SAM and SAH binding stabilize the folded form of the riboswitch in single riboswitch molecules

**Figure 6.**
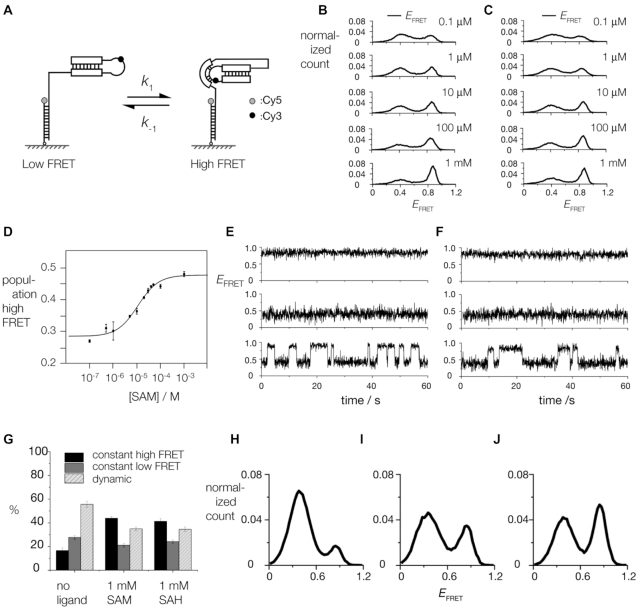
Study of ligand-induced folding of the SAM/SAH riboswitch by single-molecule FRET. (**A**) A scheme showing the probable folding of SAM/SAH riboswitch RNA. An 18 nt DNA molecule with a 3′ Cy5 acceptor (grey circle) was attached via its biotinylated 5′ terminus to a quartz slide. Cy3 donor was attached to the bulged nucleotide in the PK helix of the riboswitch, and an 18 nt 3′ DNA extension complementary to the surface-attached DNA allowed the riboswitch to be tethered to the slide. If the pseudoknot helix is not formed the fluorophores should be separated (left; low FRET efficiency) whereas in the folded structure observed in the crystal the fluorophores should be much closer (right; high FRET efficiency). (B, C) Distribution of FRET efficiencies (*E*_FRET_) for SAM/SAH riboswitch molecules as a function of SAM (**B**) and SAH (**C**) concentration. These include data from all molecules, irrespective of dynamic behaviour. (**D**) Dependence of high FRET population on SAM concentration. The data have been fitted to a simple two-state binding model. (E, F) Characteristic traces of *E*_FRET_ as a function of time recorded in the presence of 1 mM SAH (**E**) and 1 mM SAM (**F**). In both cases three representative traces are shown, illustrative of constant high FRET (top), constant low FRET (middle) and dynamic molecules (bottom) undergoing transitions between states of high and low *E*_FRET_. (**G**) Histograms showing the relative fraction of constant high FRET (black), constant low FRET (dark gray) and dynamic molecules (light grey). (H–J) Distributions of ebFRET efficiency from the dynamic molecules under different ligand conditions. (**H**) no ligand present, (**I**) 1 mM SAH and (**J**) 1 mM SAM.

On binding, SAH, SAM and related ligands become assimilated into the core of the structure, suggesting that the ligand should stabilize the pseudoknot part of the riboswitch structure. We therefore sought to test this hypothesis directly using single-molecule FRET experiments. We designed a riboswitch construct, tethered to a quartz slide by hybridization of a 3′ extension to an oligonucleotide carrying a Cy5 acceptor fluorophore at its 3′ terminus (Figure 6A). The riboswitch had a Cy3 donor attached internally within the loop region, attached to the O2′ of U20 that extends away from the PK helix in our structure. In the structure of the riboswitch adopted in the crystal, the two fluorophores should be relatively close, resulting in a high efficiency of energy transfer (*E*_FRET_). Single molecule histograms of *E*_FRET_ as a function of time show two major populations centered at 0.4 and 0.8 (Figure 6B and C). The high FRET state became more populated at higher ligand concentrations for both SAH (Figure 6B) and SAM (Figure 6C). Plots of the fraction of molecules in the high FRET state as a function of ligand concentration could be well fitted using a simple two-state binding isotherm, yielding dissociation constants (*K*_d_) of 10 μM for SAM (Figure 6D) and 6 μM for SAH. The calculated *K*_d_ values are similar to those determined from ITC in bulk solution, suggesting that attachment of the fluorescent label did not perturb the riboswitch significantly. These results are consistent with two conformations of the riboswitch. The high *E*_FRET_ state highly likely corresponds to that observed bound to ligand in the crystal. The low *E*_FRET_ state is more extended, probably lacking the PK pseudoknot.

Single-molecule traces showing *E*_FRET_ as a function of time comprised transitions between two species of high and low values. Three types of behaviour were observed (Figure 6E, F). (i) *E*_FRET_ remained low for the duration of observation, up to 1 min, (ii) *E*_FRET_ remained high for the duration of observation (iii) *E*_FRET_ fluctuated between the low and high values. All three species were observed at all ligand concentrations. However, the constant high FRET species increased in population at the expense of the dynamic species when 1 mM SAM or SAH was added (Figure 6G). The fraction of constant low FRET species was almost unaltered by ligand. The changes in relative populations of the different species was closely similar between SAM and SAH (Figure 6G), consistent with the similar dissociation constants and mode of binding.

To characterize further the dynamic species, we applied ebFRET with a two-state model to the dynamic single-molecule trajectories. For the three populations in the presence of no ligand, 1 mM SAM or 1 mM SAH, traces in excess of 50 s were collected and categorized into constant high FRET, constant low FRET, or dynamic. For the dynamic population, the regions of dynamic behavior were analyzed as two-state transitions using open-source script, ebFRET. The ebFRET algorithm uses an empirical Bayesian method to determine the FRET states and the time points at which transitions between FRET states occur using the hidden Markov model (49). The results show that ligand binding stabilizes the folded state (Figure 6H–J) and that the folding rate increases and the unfolding rate decreased when 1 mM SAM or SAH were added (Supplementary Table S4). The effect of SAM was stronger than that of SAH.

## DISCUSSION

We have determined seven crystal structures of the SAM/SAH riboswitch at high resolution bound to a number of different ligands. The majority were solved in C2, giving six crystallographically-independent molecules for each. For each structure the RNA adopts the same fold, with coaxial alignment of the P1 helix and the pseudoknot helix PK (Supplementary Figure S5). The RMSD values are given in Supplementary Table S2. The structures were unaffected by crystal contacts: the structure of the riboswitch bound to SAM was solved in space groups P312 and C2, with an all-atom RMSD of 0.26 Å. Importantly, the overall RNA trajectory in our structures solved by X-ray crystallography were in excellent agreement with that solved in solution using NMR by Wöhnert and colleagues (36) (Supplementary Figure S6A). The manner of ligand binding is also similar, with the same manner of base pairing to U23, although some other hydrogen bonds were not identified in the NMR study (Supplementary Figure S6B,C).

### Ligand binding to the SAM/SAH riboswitch

We have solved structures with six different ligands bound. All retain the adenosine moiety, with variations in the methionine chain attached to the 5′ position. The specificity of binding is largely realized through the adenosine moiety, forming four hydrogen bonds to the riboswitch RNA in each case. The adenosine forms a *trans* Hoogsteen-Watson Crick base pair with U23, and the nucleobase is stacked with RNA nucleobases on both sides. The bound adenosine moiety becomes an integral part of the helix, at the interface between the extended P1 and the PK helices. A similar manner of SAM binding was observed in the recent structure of the SAM-VI riboswitch (23) (Supplementary Figure S6D).

The adenosine moiety is located in its pocket surrounded by RNA. By contrast the methionine chain makes few interactions with the riboswitch, limited to a single hydrogen bond donated by the amide nitrogen atom. The chain extends out into the relatively open major groove of the P1 loop. The sulfur atom is 3.8 Å away from the nearest RNA atom, leaving enough space to accommodate the SAM methyl group without steric clash, and the open binding site will readily accommodate the trajectory of the chain with a planar thioether (SAH) or tetrahedral sulfonium (SAM). This is further illustrated by the structure with AMP bound, showing that the 5′ phosphate group can be accommodated in this position. In addition there is no electrostatic interaction with the positively charged sulfonium ion as there is in the SAM-II (19) and V (20) riboswitches. The structure therefore explains why this riboswitch binds both SAM and SAH with similar affinities.

A number of riboswitches bind an adenosine moiety as part of SAM (14,19,20,22), NAD^+^ (50), cobalamine (51), cyclic di-AMP (52–54) and other metabolites. This ever-expanding group provides an opportunity to compare the modes of binding of the same ligand by multiple RNA frameworks, and we have recently discussed this in detail (50). In short, the adenine nucleobase is always stacked with RNA nucleobases on one or both faces, and generally forms a base pair with the RNA. However, the manner of binding can differ in terms of the glycosidic bond angle of the adenosine ligand (*anti* > *syn*), the conformation of the base pair formed (*trans* > *cis*) and the face of the adenine that forms hydrogen bonds (Watson-Crick, Hoogsteen and sugar edges are all used). SAM adenine forms a *trans* Hoogsteen:Watson–Crick A:U pair only in the SAM/SAH riboswitch and the SAM-VI riboswitches (23). Clearly RNA evolution has found many solutions to the problem of specific binding of adenine-containing ligands.

### Structural change in the riboswitch upon ligand binding

The ligand binding site of the SAM/SAH riboswitch is created by the extension of helix P1 (i.e. the formation of the additional three base pairs that were not identified in the original secondary structure model) and the PK helix of the pseudoknot. Examination of in-line probing data of Weinberg *et al.* (16) indicates that both of these features form in response to the binding of ligand. The region C18 to U23 is reactive (i.e. flexible) in the absence of ligand, but becomes unreactive with the addition of SAM. Similarly, nucleotides C15, G16, G25 and C26 change from being reactive to protected in the presence of SAM. These data are consistent with the formation of the PK helix and the formation of the 3 bp-extension to the P1 helix respectively in response to ligand binding. NMR data also suggested that the structure was more flexible in the absence of ligand (36). The formation of these two helical sections will create the ligand binding site, and conversely ligand binding should stabilize the folded structure.

This is consistent with the single-molecule FRET data. These show clearly that the riboswitch can exist in two conformations with low and high FRET efficiency (i.e. fluorophores separated and close respectively), and that the fraction of the high FRET conformation increases with the addition of ligand (Figure 6). In our structures of the ligand-bound riboswitches, the positions of the fluorophores should be brought close together on folding (see the scheme in Figure 6A), so the folded form clearly corresponds to the high FRET state. The single-molecule FRET data show the existence of three dynamic classes of ribozyme molecules. In a previous single-molecule FRET study of the SAM-II riboswitch three similar species were observed – constant high, constant low and dynamic populations (55). In those experiments, only a 1–5% population of the dynamic traces were observed. However, in the SAM-SAH riboswitch, the dynamic population is the main population (> 50%), which implies different switching mechanisms between the two riboswitches. The molecules with constant low *E*_FRET_ are probably misfolded, and the fraction is essentially unaffected by the presence of ligand. Of greater interest are the molecules that maintain a conformation of constant high *E*_FRET_ value, and those that exhibit repeated transitions between conformations of low and high *E*_FRET_. Addition of either SAM or SAH results in a greater fraction of constant high *E*_FRET_ molecules at the expense of the dynamic ones. Complete folding requires the formation of two elements, the pseudoknot PK, and the extension of the P1 helix by the additional three base pairs. These two helices flank the position of bound ligand. We suggest that formation of the PK helix (thus generating a high *E*_FRET_ state) might occur in the absence of the P1 extension, but that this partially-folded state would be unstable, leading to the observed dynamic character. Binding SAM or SAH and formation of both helical sections is probably required to achieve the constant high *E*_FRET_ state, i.e. the structure we observe in the crystal. This model offers an explanation of the available data and will be tested in due course.

### A model for translational repression

**Figure 7.**
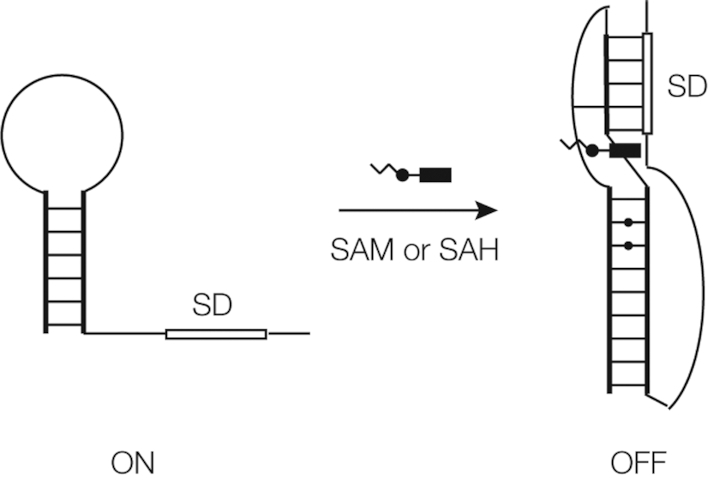
A proposed structural mechanism of translational regulation by the SAM/SAH riboswitch. In this scheme we represent the starting and end states, although intermediate folded states probably exist. In the absence of ligand we propose that the molecule predominantly exists as a simple P1 stem–loop, in which the 3′ end of the molecule that includes the Shine–Dalgarno sequence (box) is unstructured. In the presence of ligand the structure is that observed in the crystal, with the 3 bp extension to the P1 helix and the formation of the PK helix. In the absence of the ligand the Shine–Dalgarno ribosome binding site is accessible, and translation can initiate on the ribosome. However, in the folded structure this is involved in the formation of the PK helix and so inaccessible to the ribosome thus preventing the initiation of translation.

Thus all our data are consistent with stabilization of the folded structure that includes the PK helix and the P1 extension on binding ligand. This leads us to the model for translational regulation by the SAM/SAH riboswitch indicated in Figure 7. The ribosome binding site is located at the 3′ end of the RNA, and becomes one strand of the PK helix on folding. In the absence of ligand the PK helix is unstable, and therefore the stretch of RNA containing the SD sequence is available to bind to the ribosome and initiate translation (translational ON state). Binding SAM or SAH stabilizes the PK helix, so structuring the Shine–Dalgarno sequence, sequestering it and preventing initiation of translation (OFF state). This then is the molecular mechanism of riboregulation by the SAM/SAH riboswitch.

## DATA AVAILABILITY

Coordinate files 6YMM, 6YLB, 6YL5, 6YML, 6YMK, 6YMI and 6YMJ have been deposited with the PDB.

## Supplementary Material

gkaa493_Supplemental_File
