# Supplementary material for: Crystal structure and ligand-induced folding of the SAM/SAH riboswitch
Source: Nucleic Acids Res. 2020 Jun 10;48(13):7545–56. doi: 10.1093/nar/gkaa493 (PMC7367207; doi:10.1093/nar/gkaa493)
Supplement: gkaa493_Supplemental_File [file gkaa493_supplemental_file.pdf]

Structure and ligand-induced folding of the SAM/SAH riboswitch

L. Huang, T.-W. Liao, J. Wang, T. Ha and D. M. J. Lilley

SUPPLEMENTARY INFORMATION

SUPPLEMENTARY FIGURES

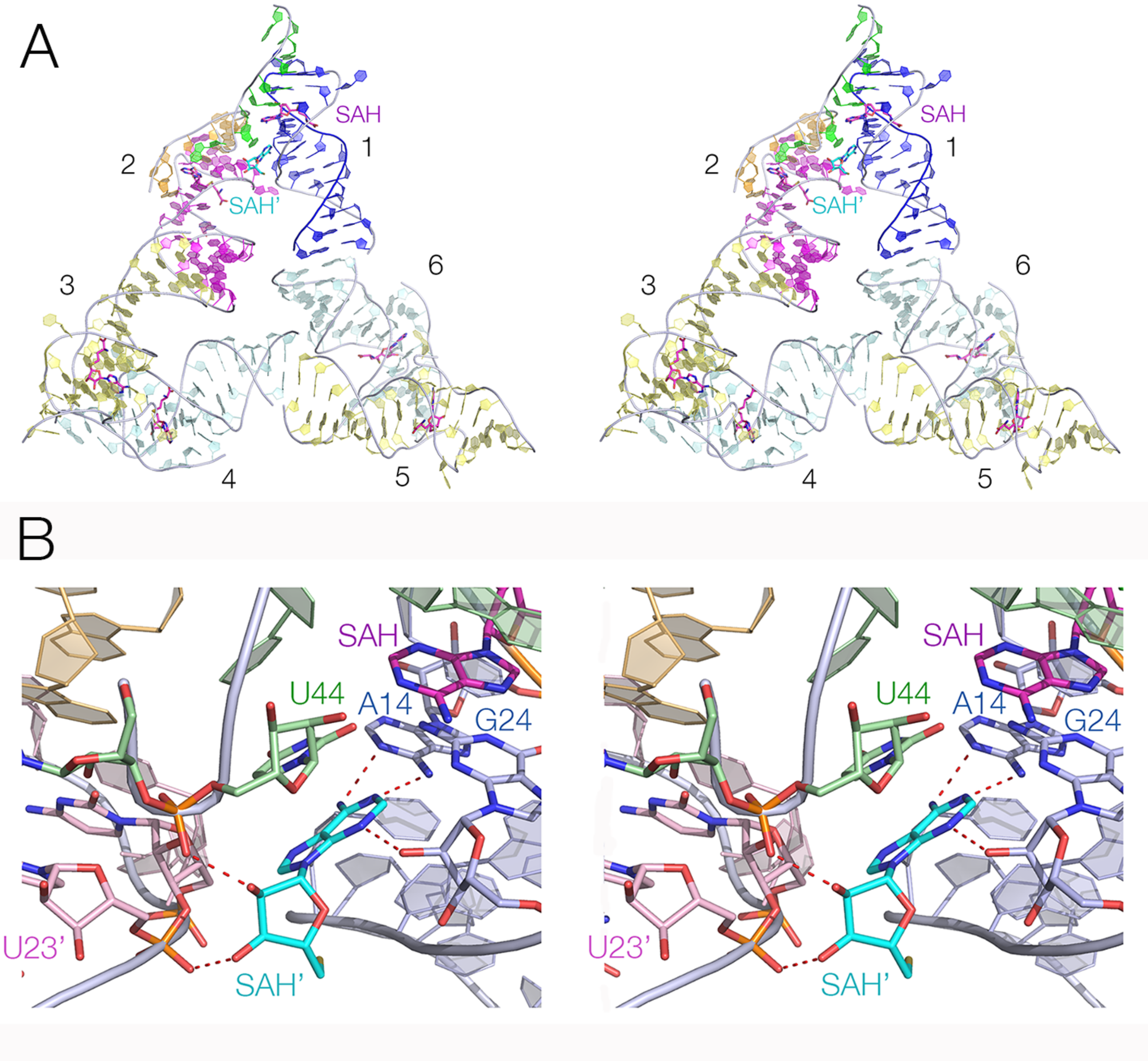

Figure S1. The packing of SAM/SAH riboswitches in the C2 crystal lattice, and the binding of a second molecule of SAH in the interface. **A**. The asymmetric unit in the crystal lattice comprises a triangular packing of six SAM/SAH riboswitches. Pairs of riboswitches are stacked in an approximately coaxial manner by their P1 helices. **B**. A second SAH molecule is bound at the apical interface between two riboswitches, and is hydrogen bonded to both. The methionine chain of the SAH is not observed in the structure.

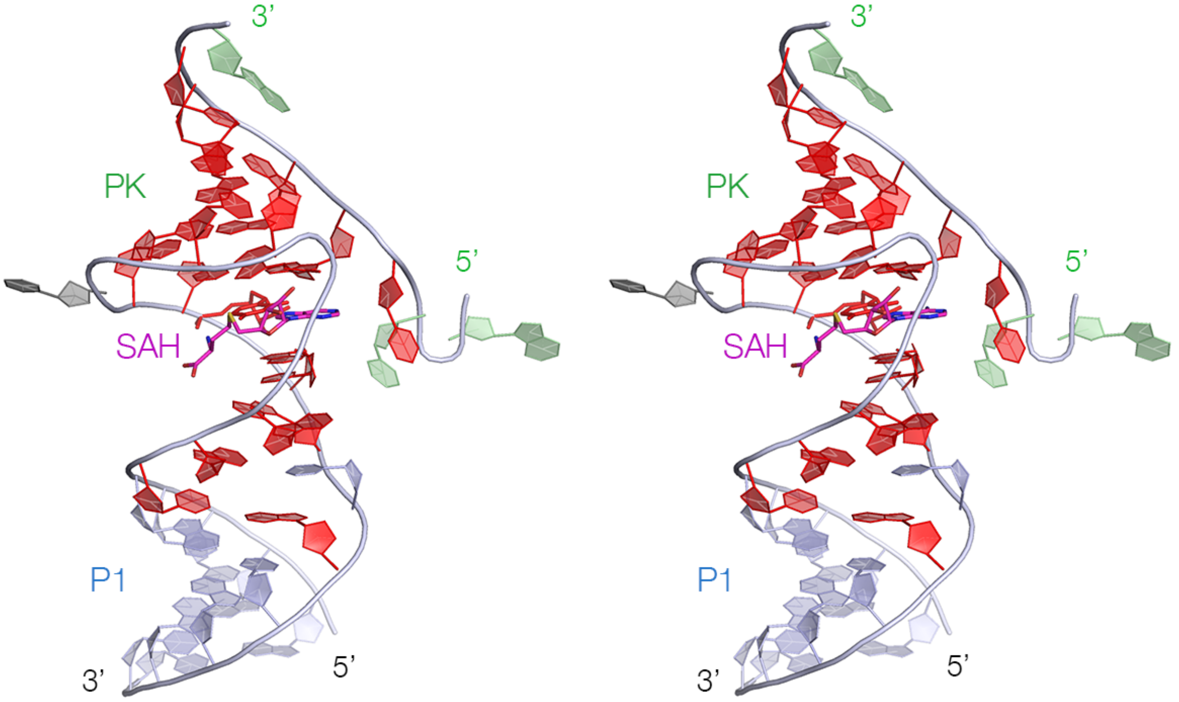

Figure S2. The location of conserved nucleotides in the structure of the SAM/SAH riboswitch. Nucleotides colored red are >97% conserved according to Weinberg, Z. *et al* (2010) *Genome biol.*, **11**, R31.

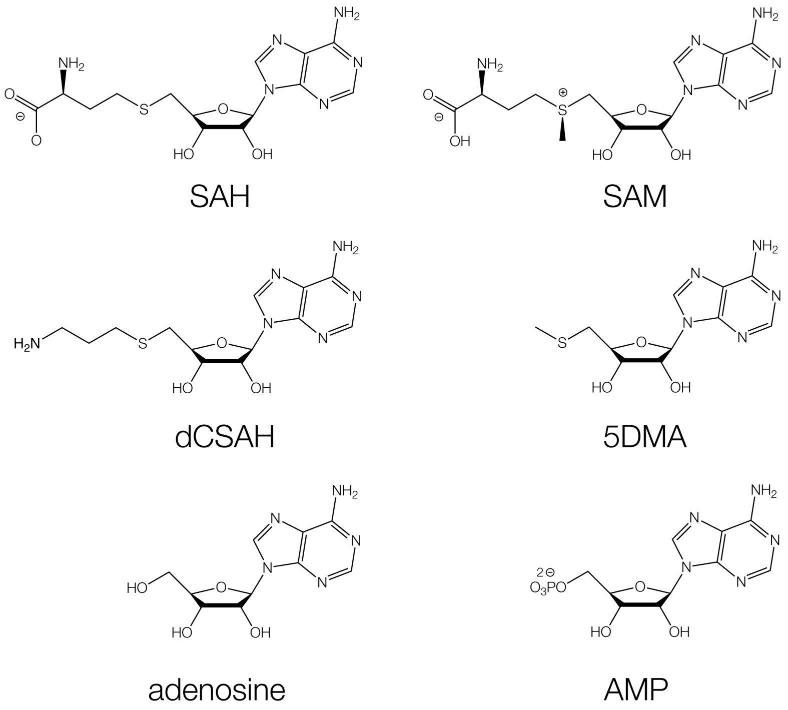

Figure S3. The chemical structures of the six ligands that have been crystallized with the SAM/SAH riboswitch in this study

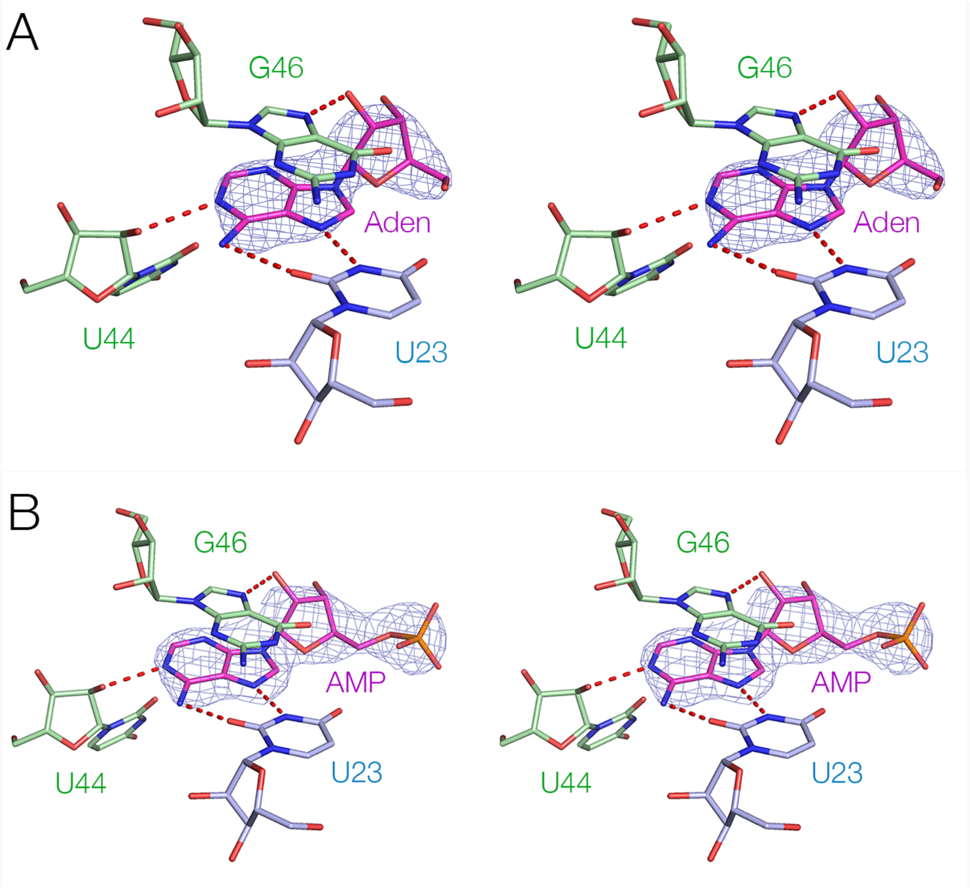

Figure S4. The binding of adenosine and AMP to the SAM/SAH riboswitch. Parallel-eye stereoscopic views of the binding site, with omit maps of electron density shown on the ligands contoured at 1.2 σ.

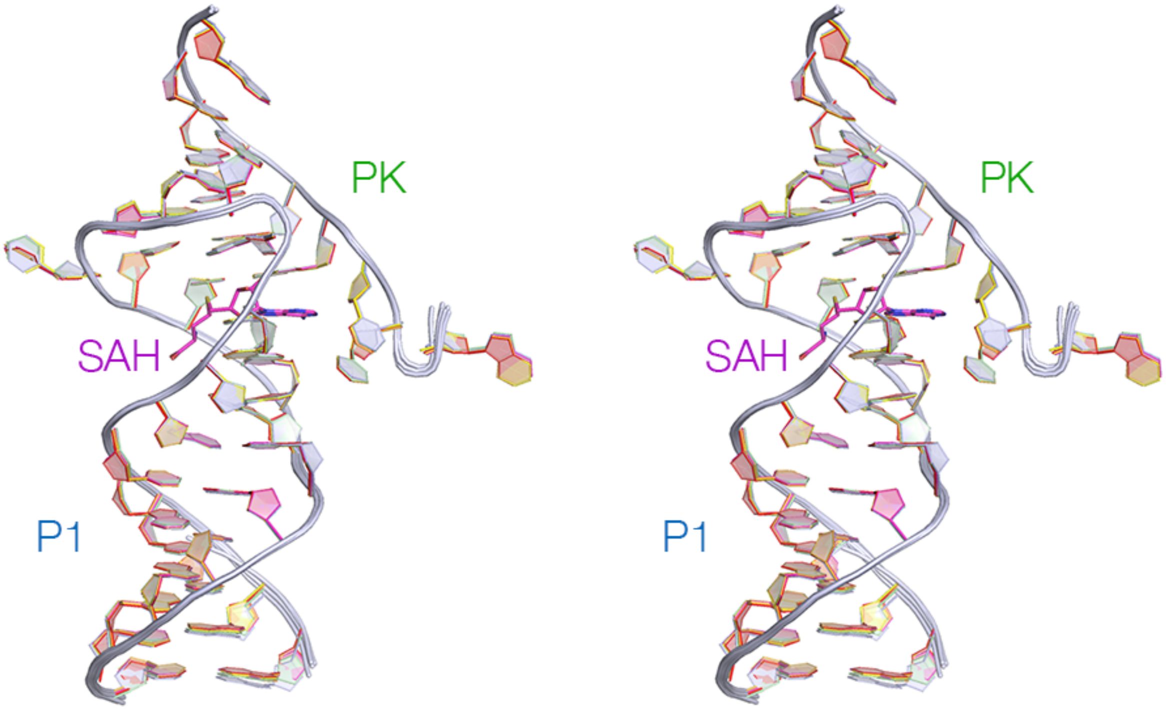

Figure S5. Superposition of riboswitch structures bound to different ligands. Parallel-eye stereoscopic views of the complete riboswitch bound to SAH (red, 6YLS), SAM in C2 (orange 6YLB), SAM in P321 (blue, 6YMM), dCSAH (green, 6YML), 5DMA (magenta, 6YMK), AMP (cyan, 6YMI) and adenosine (yellow, 6YMJ). All structures were solved in C2 except for the indicated structure in P321. In each case the AB strands are shown. RMSD values are shown in Table S2.

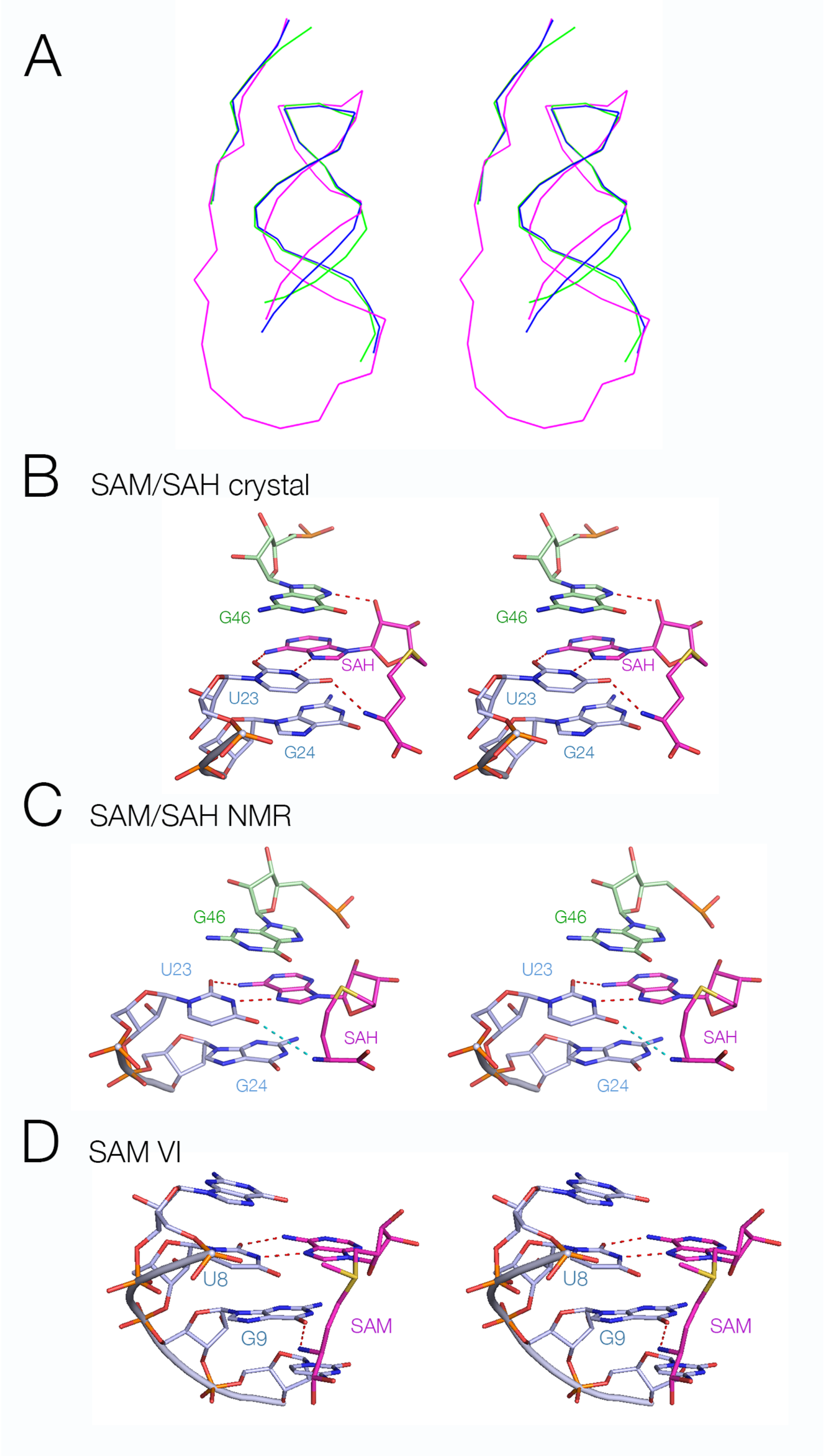

Figure S6. Superposition of the structures of the SAM/SAH riboswitch determined by X-ray crystallography and NMR, and comparison of the ligand binding sites together with that of the SAM-VI riboswitch.

A. Superposition of the SAM/SAH riboswitch backbone trajectories of the crystal structures (AB chains, blue; CD chains green) and the NMR structure (magenta). The crystal structure is the SAH-bound structure PDB ID 6YL5 and the NMR structure is PDB ID 6HAG. There is close agreement between the overall trajectories of the crystal and NMR structures.

B. SAH bound to its binding site in the crystal structure of the SAM/SAH riboswitch PDB ID 6YL5. Four of the five observed ligand-RNA hydrogen bonds are indicated in red.

C. SAH bound to its binding site in the NMR structure of the SAM/SAH riboswitch PDB ID 6HAG. Only two of the ligand-RNA hydrogen bonds observed in the crystal were identified by NMR. The distance between the SAH amide and U23 O4 (shown cyan) is 4.2 Å in the NMR structure. Weickhmann, A. K. *et al* (2019) *Nucleic Acids Res*., **47**, 2654-2665.

D. SAM bound to its binding site in the crystal structure of the SAM-VI riboswitch PDB ID 6LAS. The SAM adenine forms a *trans* Hoogsteen-Watson-Crick A:U base pair similar to that in the SAM/SAH riboswitch. Sun, A., *et al* (2019) *Nat Commun*., **10**, 5728.

SUPPLEMENTARY TABLES

ligands	SAM	SAM	SAH	Decarboxy-lated SAH	5'-Deoxy-5'-(methylthio) adenosine	AMP	Adenosine
Ligands code	SAM	SAM	SAH	DSH	MTA	AMP	ADN
PDB	6YMM	6YLB	6YL5	6YML	6YMK	6YMI	6YMJ
**Data collection**							
Space group	P312	C2	C2	P312	C2	C2	C2
Cell dimensions							
a, *b*, *c* (Å)	88.2, 88.2, 76.1	87.1, 147.9, 74.8	86.8, 147.6, 74.7	86.2, 86.2, 75.4	87.1, 147.8, 75.0	86.5, 147.4, 74.8	86.6, 147.6, 75.0
α, β, γ (°)	90 90 120	90 91.7 90	90 92.1 90	90 90 120	90 91.8 90	90 90 90	90 90 90
	SAD-Br	MR	MR	MR	MR	MR	MR
Wavelength	0.9198	0.9119	0.9119	0.9188	0.9119	0.9119	0.9119
Resolution (Å)	76.08 – 2.20 (2.24 – 2.20)	75.04 – 2.12 (2.16 – 2.12)	74.75 – 1.70 (1.73 – 1.70)	75.40 – 2.17 (2.20 – 2.17)	75.02 – 2.03 (2.07 – 2.03)	74.82 – 2.50 (2.54 – 2.50)	74.99 – 2.04 (2.08 – 2.04)
R_{merge}	0.081 (1.493)	0.087 (1.153)	0.089 (0.942)	0.095 (3.048)	0.085 (1.084)	0.111 (0.832)	0.054 (1.365)
$I / \sigma I$	17.2 (1.7)	7.4 (1.1)	7.3 (1.2)	9.4 (1.4)	7.4 (1.1)	8.3 (1.0)	11.0 (1.0)
CC (1/2)	0.97 (0.70)	0.99 (0.36)	0.99 (0.50)	0.97 (0.78)	0.99 (0.39)	0.99 (0.55)	0.99 (0.33)
Completeness (%)	99.6 (95.7)	99.9 (100)	98.4 (95.7)	100 (100)	99.7 (99.4)	99.2 (98.6)	99.8 (99.8)
Redundancy	14.2 (11.1)	3.3 (3.2)	3.4 (3.5)	9.7 (10.2)	3.3 (3.2)	3.4 (3.4)	3.3 (3.4)
**Refinement**							
Resolution (Å)	38.18 – 2.20 (2.28 – 2.20)	37.46 – 2.12 (2.20 – 2.12)	53.65 – 1.70 (1.76 – 1.70)	43.11 – 2.17 (2.25 – 2.17)	37.13 – 2.03 (2.10 – 2.03)	74.59 – 2.50 (2.59 – 2.50)	37.11 – 2.04 (2.11 – 2.04)
No. reflections	17244 (1645)	53494 (5237)	100403 (9862)	17054 (1601)	60858 (5914)	32134 (3195)	59616 (5933)
R_{work} / R_{free}	0.198 / 0.223	0.230 / 0.266	0.171 / 0.198	0.199/ 0.234	0.227/ 0.265	0.216 / 0.240	0.203 / 0.239
No. atoms							
macromolecules	1508	4519	4539	1507	4326	4533	4326
ligands	43	126	160	47	318	126	301
solvent	11	95	533	5	96	20	37
B-factors							
macromolecules	78.7	83.5	48.6	74.5	78.0	69.9	72.6
ligands	90.1	117.3	66.2	91.0	143.2	69.5	67.4
solvent	61.3	55.2	48.0	63.6	55.0	55.2	60.4
R.m.s. deviations							
Bond lengths (Å)	0.008	0.007	0.012	0.007	0.006	0.003	0.001
Bond angles (°)	1.49	1.22	1.89	1.33	0.82	0.78	0.36

*Values in parentheses are for highest-resolution shell.

Table S1. Details of data collection and refinement statistics for the crystallographic data as deposited with the PDB.

structure	chains	RMSD / Å *vs* 6YL5_SAH AB
6YL5_SAH	AB	-
	CD	0.561
	EF	0.197
	GH	0.749
	IJ	0.197
	KL	0.720
6YLB_SAM_C2	AB	0.219
	CD	0.722
6YMM_SAM_P312	AB	0.333
	CD	0.576
6YML_dSAH	AB	0.305
	CD	0.645
6YMI_AMP	AB	0.261
	CD	0.542
6YMJ_Ade	AB	0.221
	CD	0.580
6YMK_5DMA	AB	0.233
	CD	0.704

Table S2. RMSD values for superimposition of SAM/SAH riboswitches as a function of lattice position, space ground and bound ligand. All these structures were solved in C2, except that for the SAM complex that was solved in P321. Each riboswitch comprises two chains (e.g. A and B), and the asymmetric unit for C2 contains six riboswitch molecules, i.e. 12 chains A through L. We note that the CD, GH and KL chains differ slightly from AB, EF and IJ. The difference lies principally in the distal end of the P1 helix, and the core of the riboswitches are closely similar.

Ligand	RNA	*n*	ΔH kcal mol^{-1}	ΔS cal.K^{-1}mol^{-1}	ΔG kcal mol^{-1}	K_d μM	c*
SAM	wt	0.8 ± 0.1	-22 ± 1	-52	-6.4	19 ± 2	2.1
SAH	wt	0.8 ± 0.1	-23 ± 2	-56	-6.4	20 ± 2	2.0
dCSAH	wt	1.0 ± 0.1	-20 ± 1	-45	-6.7	12 ± 1	3.3
SAM	G44U	0.6 ± 0.1	-27 ± 4	-68	-6.7	13 ± 3	3.1

Table S3. Isothermal titration calorimetry of ligand binding to the SAM/SAH riboswitch at 298 K.

ligand	k_1 / s^{-1}	k_{-1} / s^{-1}	k_1 / k_{-1}
none	1.3 ± 0.14	5.0 ± 0.22	0.27
1 mM SAM	1.9 ± 0.28	3.6 ± 0.42	0.52
1 mM SAH	1.7 ± 0.23	4.5 ± 0.51	0.37

Table S4. Rates of interconversion of high and low FRET states. Rates were measured in 40 mM HEPES (pH 7.5), 100 mM KCl, 2 mM $MgCl_2$.
